# An Exploratory Analysis of Differential Tear Fluid miRNAs in Patients with Parkinson’s Disease and Atypical Parkinsonian Syndromes

**DOI:** 10.1007/s12035-025-05252-2

**Published:** 2025-08-04

**Authors:** Antonia F. Demleitner, Lucas Caldi Gomes, Lara Wenz, Laura Tzeplaeff, Dominik Pürner, Elena Luib, Lea H. Kunze, Paul Lingor

**Affiliations:** 1 Department of Neurology, School of Medicine and Health, TUM University Hospital Rechts Der Isar, Technical University of Munich, Ismaninger Str. 22, 81675 Munich, Germany; 2 DZNE, German Center for Neurodegenerative Diseases, Munich, Germany; 3 Munich Cluster for Systems Neurology (SyNergy), Munich, Germany

**Keywords:** Tear fluid, MiRNA, Biomarker, Atypical Parkinsonian syndrome, Progressive supranuclear palsy, Multiple system atrophy

## Abstract

Parkinson’s disease (PD), multiple system atrophy (MSA), and progressive supranuclear palsy (PSP) are neurodegenerative disorders diagnosed by clinical criteria with limited diagnostic specificity in early stages. Diagnostic biomarkers facilitating early and precise diagnosis are needed. Tear fluid (TF) is an easily accessible body fluid reflecting pathophysiological changes in ocular and systemic diseases. In this exploratory study, we investigate TF as a non-invasive source of disease-specific miRNAs for PD, MSA, and PSP. We demonstrate reduced TF production in PD patients. Using a real-time quantitative PCR-based array targeting 1113 miRNAs, we identified 55 exclusively expressed in PD, 35 in PSP, and 14 in MSA, respectively. Several of these have previously been identified in other biofluids. Overrepresentation analysis of target genes showed apoptotic and cell differentiation pathways as common targets. While these findings suggest that miRNA alterations in TF might reflect disease mechanisms in PD and atypical Parkinsonian syndromes, the exploratory character of the study combined with the use of pooled samples, indicates the need for further validation. The small sample size highlights the importance of follow-up studies with larger, more definitive cohorts to confirm the potential of these miRNAs as reliable biomarkers.

**Supplementary Information:**

The online version contains supplementary material available at 10.1007/s12035-025-05252-2.

## Introduction

Parkinson’s disease (PD) and atypical Parkinsonian syndromes (aPS), including multiple system atrophy (MSA) and progressive supranuclear palsy (PSP), but also corticobasal degeneration (CBD) and dementia with Lewy bodies (DLB), are neurodegenerative disorders characterized by the progressive loss of motor and cognitive functions.

While specific diagnostic criteria relying on the clinical history, physical examination, and the patient’s response to levodopa have been developed for all mentioned diseases [1–3], an overlap of symptoms is common, especially in the early stages of the disease. Furthermore, a definite diagnosis is only possible postmortem through neuropathological examination of brain tissue. Therefore, the current diagnostic approaches remain challenging and frequently result in delays and misdiagnosis [4]. This emphasizes the critical need for reliable biomarkers to facilitate early detection and more targeted interventions, ultimately enhancing disease management and patient outcomes.

MicroRNAs (miRNAs) are small, non-coding RNAs of about 22 nucleotides in length. They can regulate the post-transcriptional gene expression through binding with the 3′UTR of its target mRNA, leading to cleavage or translational repression [5]. While one miRNA regulates the expression of multiple genes, a single gene can be regulated by multiple miRNAs. Therefore, the impact of miRNAs on a given biological process is complex. In the central nervous system (CNS), miRNAs regulate key processes such as neurite outgrowth, dendritic development, neuronal differentiation, and synaptic plasticity [6]. In PD, miRNAs have been shown to play a significant role in key pathomechanisms like mitochondrial dysfunction, protein aggregation, oxidative stress, and neuroinflammation [7].

Although typically restricted to tissue, some miRNAs are released in extracellular biofluids [8]. There, their composition and levels have been shown to reflect different disease states. Growing evidence suggests that miRNA in cerebrospinal fluid (CSF) can help distinguish PD from healthy controls [9, 10]. Limited data regarding expression patterns in the different aPS is available [11, 12].

While most data on miRNAs in biofluids stems from either blood or CSF, miRNAs have also been detected in other biofluids such as saliva and tear fluid (TF) [8]. Particularly, TF has recently gained attention as a potential source of biomarkers for neurological diseases [13–17]. Although TF is an ultrafiltrate from blood, the lacrimal gland, through the innervating parasympathetic nerves, receives input from regions in the brain stem—areas commonly affected in most neurodegenerative disorders. Interestingly, reduced TF production has been demonstrated in a range of neurodegenerative diseases, supporting the widespread involvement of the lacrimal system [18]. TF could therefore serve as a valuable, non-invasive, and easily accessible bioliquid for early detection and monitoring of PD and other neurodegenerative diseases. Indeed, several studies report changes in established biomarkers of neurodegenerative diseases such as PD, Alzheimer’s dementia (AD), Creutzfeldt-Jakob disease, and Huntington’s disease [14, 15, 19–21].

Data on miRNAs in the TF of patients with neurodegenerative diseases is limited. Changes in the expression of miRNAs associated with amyloid beta production and inflammation in TF of transgenic mice mimicking AD have been linked to concomitant neurodegeneration [22]. Kenny et al. have demonstrated high concentrations of miRNA in TF with a significant difference in total miRNA levels between AD and healthy controls. Moreover, specific miRNAs have been identified to serve as potential biomarkers [16]. However, the role of miRNA in TF of PD and aPS remains unexplored.

**Fig. 1:**
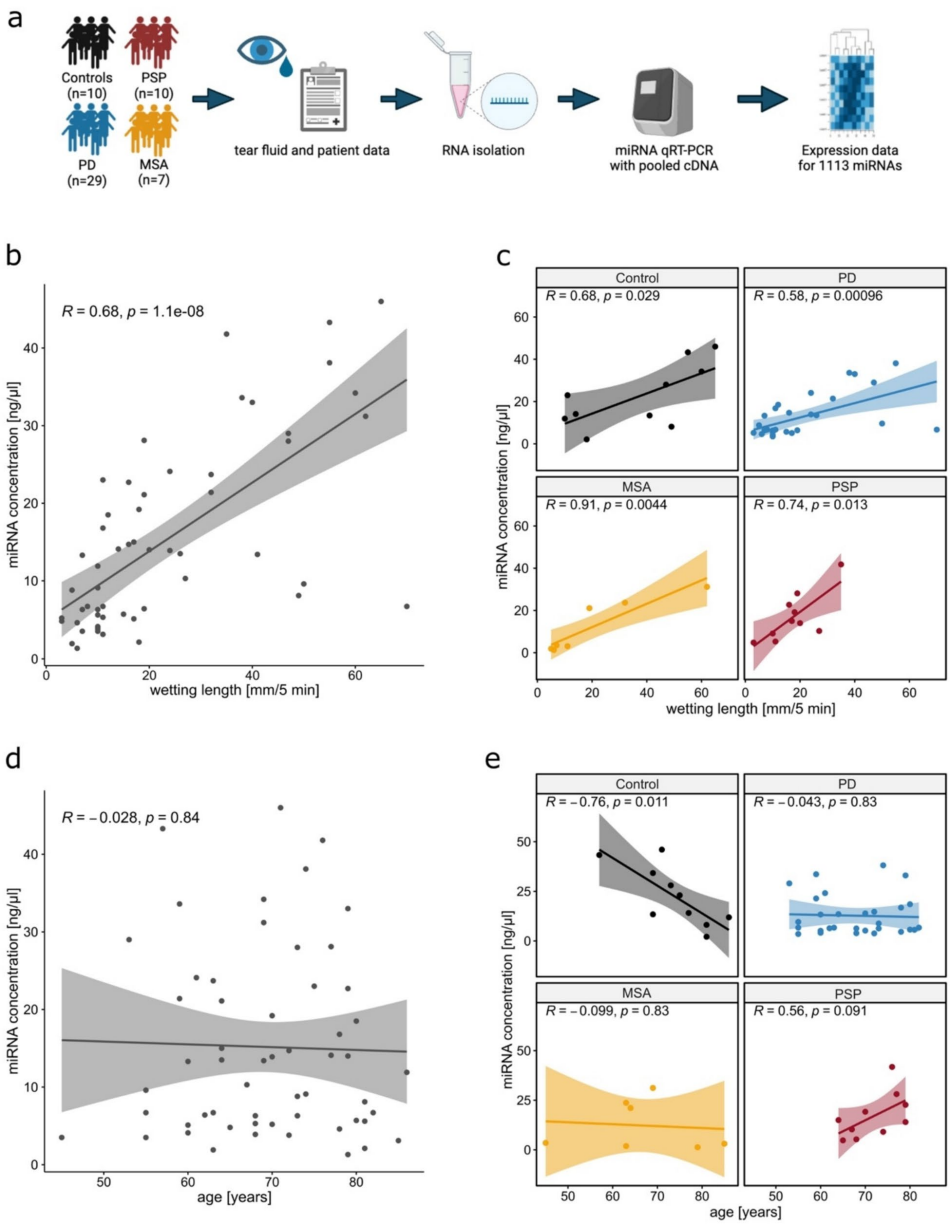
Correlation of miRNA concentrations in tear fluid with clinical parameters. **a** Overview of the experimental setup (created with Biorender). TF and patient data were collected from patients and controls. After RNA isolation, Real-Time quantitative PCR (RT-qPCR) was performed and expression data analyzed. **b** Correlation of miRNA concentration and wetting length in the overall cohort. **c** Correlation of miRNA concentration and wetting length within the subgroups of controls (black), PD (blue), MSA (orange), and PSP (red). **d** Correlation of miRNA concentration and age in the overall cohort. **e** Correlation of miRNA concentration and age within the subgroups of controls (black), PD (blue), MSA (orange), and PSP (red). PD, Parkinson’s disease; MSA, multiple system atrophy; PSP, progressive supranuclear palsy. Pearson’s correlation (Coefficient = R) was used for analyses. Data are depicted as a regression line with a 95% confidence interval and individual data points. Panel **a** was created in BioRender (Demleitner, A. (2025) https://BioRender.com/727vhvc)

This study aimed to investigate the expression patterns of miRNA in TF of PD, MSA, and PSP patients compared to healthy controls to gain insight into miRNAs as possible biomarkers in diagnosing Parkinsonian syndromes. For this, we performed an RT-qPCR-based analysis of the miRNAome of PD, MSA, and PSP as well as control TF samples using pooled cDNA samples (Fig. 1a). The results of our exploratory analysis show distinct differences between groups.

## Results

TF was collected using Schirmer test strips from 56 patients. Ten of these were healthy control patients without evidence of neurodegenerative disease, and 29 were patients with probable or clinically established PD (mean Hoehn and Yahr stage 2.2 +/− 1). In addition, we included 7 patients with either possible or probable MSA, of which 4 were of the cerebellar and 3 of the Parkinsonian subtype, and 10 patients with either possible or probable PSP were included. All patients were well matched regarding age, sex, concomitant eye diseases, or medication (Table 1). Crucially, the use of systemic medications with anticholinergic effects was evenly distributed between the groups. Disease duration, however, was significantly shorter in both MSA and PSP, compared to PD (mean 8.3 +/− 0.7 years in PD, 1.9 +/− 0.9 years in MSA, and 2.7 +/− 1.6 years in PSP, p_ANOVA_ = 0.005, p_Posthoc-PSP_ = 0.02, p_Posthoc-MSA_ = 0.03). Importantly, wetting length (WL) of the Schirmer test strips was significantly shorter in the PD but not the PSP and MSA group compared to the control group when correcting for age in a multiple linear regression model (37 +/− 22 mm/5 min in control, 21 +/− 17 mm/5 min in PD, 20 +/− 21 mm/5 min in MSA, 18 +/− 9 mm/5 min in PSP) (*p* = 0.02, estimate − 10.73, 95% confidence interval [− 19.39, − 2.07]).

**Table 1:** Characteristics of the study population

		Controls	PD	MSA	PSP	*p*
Patients	*N*	10	29	7	10	
Sex (female)	*N (%)*	3 (30%)	5 (17%)	2 (29%)	6 (60%)	**0.89**^**a**^
Age (years)	*Mean* + */− SD*	74 +/* − *8.2	68 +/* − *9.1	67 +/* − *13	72 +/* − *5.8	**0.20**^**b**^
	*Median (min–max)*	74 (57–86)	68 (53–82)	64 (45–85)	72 (64–79)	
Clinical data						
Disease duration (years)	*Mean* + */− SD*	NA	8.3 +/* − *0.7	1.9 +/* − *0.9	2.7 +/* − *1.6	**0.005^**b**^
	*Median (min–max)*	NA	6.4 (0.1–23)	1.5 (0.9–3.1)	3.0 (0.3–5)	
Hoehn & Yahr Stage	*Mean* + */− SD*	NA	2.2 +/* − *1			
Ophthalmic data						
Eye disease (yes)	*N (%)*	3 (30%)	8 (28%)	1 (14%)	2 (20%)	**0.91**^**a**^
Eye medication (yes)	*N (%)*	4 (40%)	7 (24%)	1 (14%)	1 (10%)	**0.49**^**a**^
Anticholinergic medication (yes)	*N (%)*	1 (10%)	7 (24%)	1 (14%)	2 (20%)	**0.18**^**a**^
	*Missing (%)*	2 (20%)	0 (0%)	0 (0%)	0 (0%)	
Wetting length (mm/5 min)	*Mean* + */− SD*	37 +/* − *22	21 +/* − *17	20 +/* − *21	18 +/* − *9	*0.01^**c**^
	*Median (min–max)*	44 (10–65)	12 (3–70)	11 (5–62)	18 (3–35)	
miRNA concentration (ng/µl)	*Mean* + */− SD*	22 +/* − *15	13 +/* − *10	12 +/* − *13	17 +/* − *11	**0.52**^**a**^
	*Median (min–max)*	19 (2.1–46)	6.7 (3.5–38)	3.5 (1.3–31)	14 (4.8–42)	

Continuous data are presented as either median (minimum–maximum) or mean (+/− standard deviation). Categorical data are presented as absolute numbers (percentages). *PD* Parkinson’s disease, *MSA *multiple system atrophy, *PSP* progressive supranuclear palsy. a Fisher’s exact test. b One-way ANOVA with Tukey post hoc testing. c Multiple linear regression correcting for age.*p < 0.05; **p < 0.01

### miRNA Concentration Correlates with Wetting Length

We next isolated miRNA from the TF eluate and quantified its concentration. No significant difference between the groups was observed (Table 1). Correlating the miRNA concentration to clinical parameters, a significant and strong correlation with WL was observed in the overall cohort (*R*_P_ = 0.68, *p* < 0.001, 95% CI [0.50, 0.80]) (Fig. 1b). This effect was persistently observed in each of the individual patient groups, as well (control *R*_P_ = 0.68, *p* = 0.029, 95% CI [0.10, 0.92], PD *R*_P_ = 0.58, *p* < 0.001, 95% CI [0.27, 0.78], MSA *R*_P_ = 0.91, *p* = 0.004, 95% CI [0.50, 0.99], PSP *R*_P_ = 0.74, *p* = 0.013, 95% CI [0.22, 0.94]) (Fig. 1c). Age at sampling and miRNA concentration did not show any significant correlation in the overall cohort (Fig. 1d). However, a strong negative correlation was found in the control group (*R* =  − 0.76, *p* = 0.011, 95% CI [− 0.94, − 0.24]). In contrast, no trend was observed for PD, MSA, and PSP (Fig. 1e). Lastly, we evaluated the relationship of disease duration and miRNA concentration. Combining all disease groups, PD, MSA, and PSP, no significant correlation was observed. Exploring the effect in the subgroups, no correlation was seen for PD and PSP, whereas a strong positive correlation was seen for MSA (*R* = 0.84, *p* = 0.038, 95% CI [0.08, 0.98]) (Supp. Fig. S1).

### PCR-Profiling of miRNAs in the Tear Fluid of Patients with PD and aPD

**Fig. 2:**
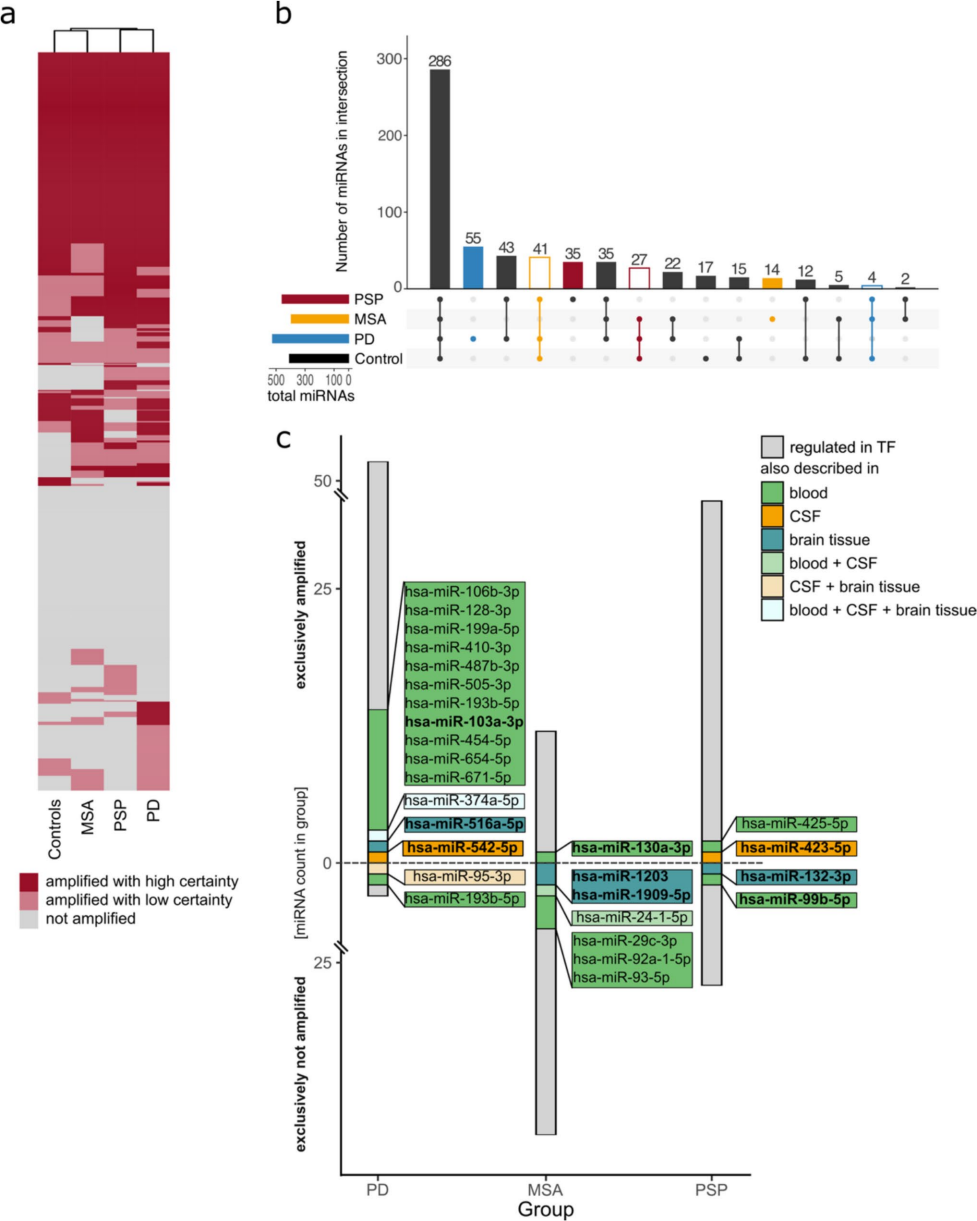
Expression analysis of the miRNA RT-qPCR screen. **a** Heatmap using unsupervised hierarchical clustering showing expression data for all 1113 quantified miRNAs. miRNAs were assigned to different groups of probability of expression: miRNAs with both technical replicates showing a CT value < 40 and an SD < 5 were considered to be “amplified with high certainty” (dark red), miRNAs not amplified in both replicates were classified as “not amplified” (grey); all else were considered “amplified with low certainty” (light red). **b** UpSetR plot showing the intersections of miRNAs between the different groups. miRNAs present in the intersects are amplified in both technical replicates with a cycle threshold of < 40 and a standard deviation of < 5. Intersections representing miRNAs exclusively amplified in each disease group are filled in color. In contrast, intersections representing miRNAs found in all but the disease group are outlined in color (blue = PD, orange = MSA, red = PSP). **c** Stacked bar graph representing the number of miRNAs in each disease intersection (consisting of the exclusively and exclusively not amplified miRNAs). The smaller bar to the right of the grey bar representing all miRNAs in the intersection shows the number and detailed annotation of miRNAs described for the respective disease in literature previously. PD, Parkinson’s disease; MSA, multiple system atrophy; PSP, progressive supranuclear palsy

After isolation of miRNAs, we quantified the miRNA levels of the pooled control, PD, MSA, and PSP samples using an RT-qPCR-based miRNA profiling kit. Of all 1113 quantifiable miRNAs, 286 were found in all groups, whereas 244 miRNAs were not found in any group. Unsupervised hierarchical clustering of the expression status of all quantified miRNAs revealed a clustering of the PD and PSP groups, as well as the MSA and control groups (Fig. 2a). Next, we aimed to identify miRNAs that were uniquely identified in each of the disease groups. For this, all miRNAs exclusively amplified with high certainty in the respective groups or exclusively amplified in all other groups, but not the disease group itself, were analyzed using an UpSetR plot (Fig. 2b). Fifty-five miRNAs were exclusively amplified with high certainty in the PD group, whereas 4 miRNAs were exclusively amplified with high certainty in all groups but the PD group. In the MSA group, 14 miRNAs were exclusively amplified with high certainty, and 41 miRNAs were exclusively amplified with high certainty in all other groups except for the MSA group. 35 miRNAs were exclusively amplified with high certainty in the PSP group, whereas 27 miRNAs were exclusively amplified with high certainty in all groups but the PSP group (a detailed listing of all miRNAs in the intersections is available in Supplementary Table S1). The annotated names from the profiling kit were converted to the current annotation in miRBase (v22), and only currently annotated miRNAs were used for further analysis.

### Presence of TF miRNA in Other Biofluids

To understand whether the miRNAs identified have been previously described in other biomaterials or are potentially specific for tear fluid, we compared our findings to data obtained in other biofluids. A detailed listing of all literature used for this comparison can be found in Table 2 (for a detailed description of methodological aspects of the search, see the “Literature search” section). While approximately one-third of the miRNAs did not show any differential expression in the respective disease groups in other biofluids before, some miRNAs have been shown to be differentially regulated (Fig. 2c). For the PD intersections, 56 of the 59 miRNAs identified as being either exclusively present or absent in PD with the profiling kit were annotated in the current version of miRBase (v22). Of those, 40 were not previously described as significantly altered in PD. 12 were previously described in blood, hsa-miR-542-5p was previously described in CSF, and hsa-miR-516a-5p in brain tissue. Hsa-miR-95-3p and hsa-miR-374a-5p have been identified to be altered in CSF and brain tissue, while the latter was also described in blood. 53 of the 55 miRNAs found in the MSA intersections are currently annotated. Of these, 46 have not been shown to be altered in MSA before. Hsa-miR-130a-3p, hsa-miR-29c-3p, hsa-miR-92a-1-5p, and hsa-miR-93-5p have been depicted differently expressed in blood, while hsa-miR-1203 and hsa-miR-1909-5p were discriminative in brain tissue. Hsa-miR-24–1-5p was shown to be altered in blood and CSF. Lastly, while we identified 62 miRNAs in the PSP intersects, only 60 of them were annotated in miRBase. Of these, 56 have not been described as altered in other biofluids. Hsa-miR-425-5p and hsa-miR-99b-5p were previously shown to be altered in blood. Hsa-miR-423-5p was identified to be changed in CSF and hsa-miR-132-3p in brain tissue. Taken together, most previously described miRNAs have been shown in blood, while only two and four have been identified in CSF and brain tissue, respectively. Only three miRNAs, two of them belonging to the group of miRNAs identified in PD, have been described in more than one biomaterial. Most miRNAs, namely 16, have been described in PD, while only seven and four have been identified in MSA and PSP, respectively.

**Table 2:** MicroRNAs in the intersections of the cohort

Group	miRNA	Blood	CSF	Brain Tissue
PD	hsa-miR-106b-3p	Xie 2022		
PD	hsa-miR-128-3p	Ravanidis 2020, Braunger 2024		
PD	hsa-miR-193a-3p	Dong 2016		
PD	hsa-miR-199a-5p	Martins 2011, Li 2024		
PD	hsa-miR-410-3p	Ravanidis 2020		
PD	hsa-miR-487b-3p	Kern 2021		
PD	hsa-miR-505-3p	Khoo 2012, Yao 2018		
PD	hsa-miR-193b-5p	Baghi 2021		
PD	hsa-miR-103a-3p	Schwienbacher 2017, Serafin 2015, Soto 2023		
PD	hsa-miR-454-5p	Cardo 2013		
PD	hsa-miR-654-5p	Cai 2021, Hou 2023		
PD	hsa-miR-671-5p	Uwatoko 2019, Khoo 2012		
PD	hsa-miR-542-5p		Mo 2016	
PD	hsa-miR-516a-6p			Hoss 2016, Chatterjee 2017
PD	hsa-miR-95-3p		dos Santos 2018	Briggs 2015
PD	hsa-miR-374a-5p	Martins 2011, Tong 2022	Tong 2022	Briggs 2015
MSA	hsa-miR-93-5p	Pérez-Soriano 2020		
MSA	hsa-miR-92a-1-5p	Kume 2017		
MSA	hsa-miR-29c-3p	Vallelunga 2014		
MSA	hsa-miR-130a-3p	Kume 2017		
MSA	hsa-miR-1203			Wakabayashi 2016
MSA	hsa-miR-1909-5p			Wakabayashi 2016
MSA	hsa-miR-24–1-5p	Vallelunga 2014, Kume 2017	Marques 2017	
PSP	hsa-miR-425-5p	Manna 2021, Ramaswamy 2022		
PSP	hsa-miR-99b-5p	Ramaswamy 2022		
PSP	hsa-miR-423-5p		Nonaka 2022	
PSP	hsa-miR-132-3p			Smith 2011

miRNAs reported to be significantly changed between the disease groups and control in other biofluid studies, sorted by group as indicated in the first column and by color (blue =  PD, orange = MSA, red = PSP)

*PD* Parkinson’s disease, *MSA* multiple system atrophy, *PSP* progressive supranuclear palsy, *CSF* cerebrospinal fluid

To further explore the potential origin of all not previously described miRNAs, we utilized a tissue expression database approach using the DIANA-miTED online tool. Interestingly, mostly (35 out of 40 for PD, 35 out of 46 in MSA, and 52 out of 57 in PSP) have been identified in brain tissue before. For the remaining miRNAs, we employed literature research querying for the terms “tear fluid” or “tear” or “lacrimal”, which did not yield any results.

### Overrepresentation Analysis of the Unique Intersections of miRNAs

**Fig. 3:**
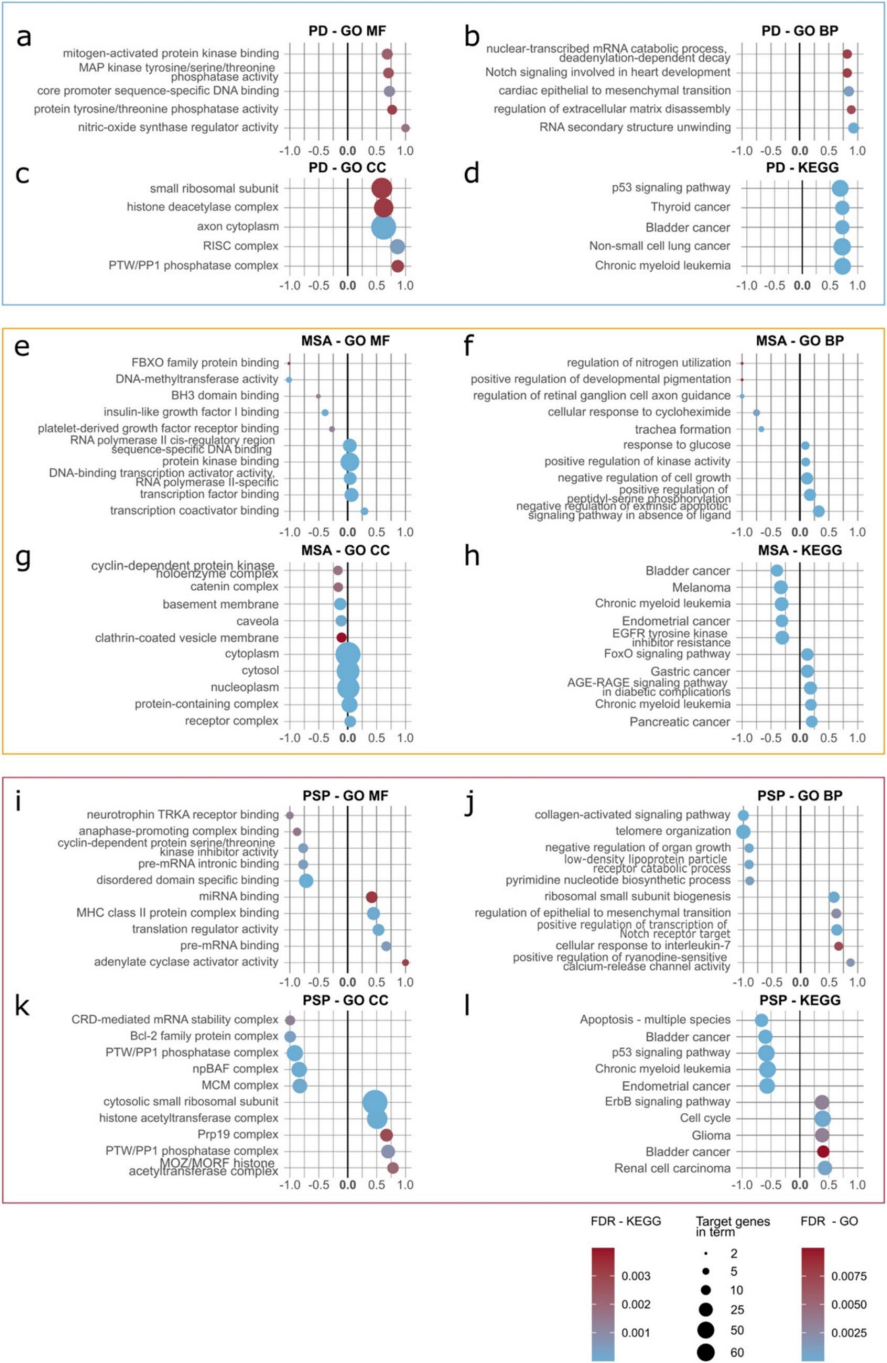
Overrepresentation analysis of the miRNAs in the PD, MSA and PSP intersections. The top 5 terms sorted by enrichment ratio with FDR < 0.01 and at least 5 miRNAs with targets in the terms are depicted. For each disease group (PD = **a**–**d** (blue box), MSA = **e**–**h** (orange box), PSP = **i**–**l** (red box), GO MF, GO BP, GO CC, and KEGG are shown (from left to right, top to bottom). Enrichment ratio (indicated below each graph) was calculated by dividing the number of enriched genes in the term by the total number of genes in the term. Negative values were assigned to terms derived from the analysis of the intersection of miRNAs exclusively not found in the disease group, positive values to those terms derived from the analysis of the intersection of miRNAs exclusively found in the disease group. Sizes of the dots represent target genes in the term, and color represents the FDR. PD, Parkinson’s disease; MSA, multiple system atrophy; PSP, progressive supranuclear palsy; GO, Gene Ontology; MF, Molecular Function; BP, Biological Process; CC, Cellular Component; KEGG, Kyoto Encyclopedia of Genes and Genomes pathways

To better inform about the potential function of the miRNAs identified in TF, we performed an exploratory overrepresentation analysis (ORA) using the DIANA miRPath online tool (v4). miRNAs that were exclusively identified in the respective disease groups were analyzed separately from those exclusively absent in one of the respective groups (Fig. 3). Negative enrichment ratio values were assigned to exclusively absent miRNAs and positive values to exclusively present miRNAs. Semantic similarity analysis (using REVIGO) was then performed to reduce redundancy and summarize the complete list of terms.

After filtering, the GO term analysis for the PD intersections revealed only significantly enriched terms pointing to the *mitogen-activated protein kinase (MAPK) pathway* in the terms annotated to the molecular function (MF) category, supported by the REVIGO analysis, which summarized the enriched terms under *protein kinase activity* (Fig. 3a, Supplementary Fig. S2, Supplementary Data S2). The MAPK pathway has been associated with cell proliferation, differentiation, and survival. Exploring the REVIGO analysis in detail, several terms belonging to in-utero embryonic development are listed in the biological process (BP) category, e.g., nervous system development, axon guidance, and generation of neurons (Supplementary Fig. S2, Supplementary Data S2). Additionally, several other terms are summarized under the terms *regulation of cell population proliferation* and *apoptotic process* (Supplementary Fig. S2, Supplementary Data S2).

For the miRNAs exclusively found in MSA, several apoptosis-related terms were enriched in the BP category, such as *negative regulation of cell growth* and *negative regulation of extrinsic apoptotic signaling pathway* (Fig. 3f, h), which was also complemented by the REVIGO analysis (Supplementary Fig. S3, Supplementary Data S2). Similar to the analysis in the PD group, *heart development* was another overarching term in the semantic similarity analysis of these BP terms, which contained many terms belonging to developmental pathways such as *nervous system development* (Supplementary Fig. S3, Supplementary Data S2).

Lastly, we analyzed the annotated terms of the ORA of the PSP miRNA intersections. Here, looking into the MF terms, *neurotrophin TrkA receptor binding *a term belonging to a family of terms encompassing many neurotrophic pathways associated with neuronal development was one of the most enriched ones among the MF terms enriched in the exclusively absent miRNAs (Fig. 3i). Interestingly, some terms associated with immune system-related functions were among the top terms enriched in the exclusively present miRNAs, such as *MHC class II protein complex binding* in the MF terms and *cellular response to interleukin-7* in the BP terms (Fig. 3j). Semantic similarity analysis revealed apoptotic pathways summarized under the term of *apoptotic process* in the BP category of the exclusively present miRNAs (Supplementary Fig. S4, Supplementary Data S2). Interestingly, *microtubule-based process* was also among the terms enriched in this category (Supplementary Fig. S4, Supplementary Data S2).

## Discussion

In this pilot study, we provide a first description of the miRNAome in the TF of a cohort of patients with PD and the atypical Parkinsonian syndromes MSA and PSP. The WL of the Schirmer test strips was significantly reduced in the PD group compared to controls, and this finding was previously observed in other cohorts [18, 20]. Predominantly postganglionic autonomic dysfunction is part of the pathology in PD [23]. While predominantly preganglionic autonomic dysfunction is a hallmark symptom in MSA [24], the impairment of the autonomic nervous system in PSP is less understood [25]. Thus, while WL did not differ significantly in the MSA and PSP groups compared to the control group, it is less apparent whether this might be due to the small sample size of these groups or other contributing factors.

We show a strong positive correlation of miRNA concentration with WL across all disease groups, while no difference in concentration between the disease groups was observed. A positive correlation between protein concentration in TF and WL has been observed in several studies [20, 26]. Interestingly, another study looking into the miRNA profiles of TF in patients with AD describes significantly increased miRNA concentration in AD, while the WL was not significantly different from the control group [16]. Dysregulation of specific miRNAs has been shown in a wide range of pathological processes. Whether these effects stem from a particular group of miRNAs with major differences in expression or from a global impact on miRNA production is yet unexplored.

We further explored the specific miRNAs expressed in the TF of our cohort. For this, we used an RT-qPCR-based approach and pooled samples to account for the relatively low number of TF samples. Normalization in TF samples is challenging, as commonly used normalization targets are unavailable. We therefore chose to categorize the data based on raw CT values into 3 groups: amplified with high certainty, amplified with low certainty, and not amplified. Our TF analyses revealed 286 miRNAs in all conditions, which aligns with previous findings exploring the total miRNAome in TF [8, 27]. We searched the literature to identify miRNAs already described in the conditions. In contrast to previous literature, we identified 40 miRNAs that have not yet been described in PD, and 46 and 56 miRNAs that are described for the first time in MSA and PSP, respectively. The fact that more miRNAs in the PD cohort have been previously described might result from more available studies concerning PD. The large number of miRNAs previously reported from blood samples may also reflect a publication bias, as most of the studies so far have looked into miRNAs in blood [10, 28]. However, as TF is mainly an ultrafiltrate from blood and studies have shown similarities between the miRNAome of TF and plasma when comparing biofluids from the same individual [29], the comparably large overlap of differentially expressed miRNAs found in TF and blood could also result from the close connection between both biofluids. The second largest overlap was observed with miRNAs previously described in brain tissue. As the lacrimal gland receives parasympathetic innervation from the brain stem, these miRNAs could have entered the TF through neuronal vesicles via anterograde synaptic transport. Exploring potential origins of the remaining, not previously described miRNAs, most were identified in brain tissue before. Data on the expression of miRNAs in tear fluid, however, is limited.

Although the miRNAs we identified in TF are extracellular and likely do not exert their known regulatory functions on RNA, overrepresentation analysis of their predicted target genes offers valuable insights into potential disease mechanisms regulated by these miRNAs. It is important to emphasize that the identified mechanisms do not represent functional effects, but rather indirect associations with disease processes. Additionally, it is important to point out that these miRNAs are not directly serving as intercellular regulators but rather potential markers of these processes. Further, these analyses were intended to be exploratory, as the miRNAs identified have not yet undergone statistical validation. The analysis may, however, offer valuable insights for further investigation. Terms comprising functions in *cell death and differentiation,* such as *angiogenesis* and in utero* embryonic development,* are shared among the disease groups. They all encompass important cell cycle-related proteins that play a crucial role in the survival of mature neurons and neuronal apoptotic processes [30, 31]. Cell death is an essential hallmark of all neurodegenerative diseases, including PD, MSA, and PSP, and is closely linked to the activation of apoptotic processes [32, 33]. Importantly, all these processes were identified in all studied disease groups, highlighting their importance for neurodegeneration in general. Some processes, however, were only associated with specific entities. For example, terms belonging to the MAPK pathway were enriched in the PD group. Although the MAPK pathway has also been implicated in cell death and apoptosis, evidence also suggests roles in axonal growth and guidance as well as oxidative stress in neurodegenerative diseases in general, including PD [34]. In the analysis of the PSP-associated miRNAs, neurotrophin signaling-related terms were highlighted. Neurotrophic factors have been linked to the survival of neurons in other neurodegenerative diseases [35]. Interestingly, the semantic similarity analysis identified *microtubule-based process* as an overarching term enriched in the miRNAs exclusively found in PSP. Notably, mutations in microtubule-associated protein tau (MAPT) are found in familial PSP cases, and MAPT aggregations are a hallmark of the disease [36]. Further, microtubule defects have been shown in mesenchymal stromal cells of patients with PSP, suggesting the involvement of microtubule-associated processes in the disease pathology of patients with sporadic disease [37]. The low number of discriminative terms for the MSA groups might be attributable to the bigger heterogeneity, as well as the lower number of patients in this subgroup: more PD and PSP patients were diagnosed as clinically established according to the respective diagnostic criteria compared to the MSA group.

Our study has apparent limitations: Given the rarity of MSA and PSP, our retrospective analysis had a limited number of patients. As a result, we included individuals with limited diagnostic certainty of MSA and PSP in the study. It is important to note that a definite diagnosis can only be established through post-mortem neuropathological analysis, which was not available in this study. Further, we had to pool individual samples because of the relatively low number of samples and the low relative amount of miRNA from each patient. Albeit this limits the ability to draw robust conclusions on the individual patient level, the PCR-based analysis of the pool was sufficient to identify miRNAs present or absent in each of the studied conditions as an exploratory approach. Sample pooling in a hypothesis-generating approach, as is this study, is a valid option with respect to balancing budgetary constraints and low concentration of the studied target molecule, when the signal is close to or below the detection threshold [38, 39]. Furthermore, we did not analyze absolute expression levels of the miRNAs but instead converted them into categorical data to circumvent normalization problems due to the lack of established normalization miRNAs. Consequently, because of the limited sample size and available samples, it was out of the scope of this study to validate the identified candidates. To establish their role as reliable biomarkers, subsequent studies with a larger number of subjects, as well as exploring the possibilities of enhancing the sensitivity of the detection methods or the concentration of the samples, will be needed to validate the identified candidates on an individual level and for stratification of single patients.

Taken together, our exploratory study provides evidence for altered expression of miRNAs in the TF of patients with PD, MSA, and PSP. It highlights the potential of TF as an easily accessible, non-invasive biomarker fluid suitable for longitudinal assessments. Further studies in bigger cohorts and individual samples are needed to confirm and expand our preliminary findings.

## Materials and Methods

### Study Design and Participants

This retrospective, monocentric cohort compiles samples collected at the Department of Neurology of the TUM University Hospital rechts der Isar in Munich, Germany, from September 2019 to February 2021. TF was collected from 46 patients with either PD or atypical Parkinsonian disorders, namely MSA or PSP, as well as control patients without signs of neurodegenerative disease. The detailed characteristics of the cohort are given in Table 1. Patients were included if the disease was at least probable according to the respective Movement Disorder Society clinical diagnostic criteria [1–3]. No other inclusion or exclusion criteria regarding age, sex, disease duration, concomitant diseases, or medication were applied. Written informed consent was obtained from all participants. The study complies with the Declaration of Helsinki and was approved by the Ethics Committee of the Technical University of Munich, School of Medicine (approval numbers: 9/15S, 2021–473-S-KH).

### Tear Fluid Sampling and Sample Preparation

TF collection was performed as previously published [18]. In brief, we employed a standardized protocol of the Schirmer test using uncolored filter strips (Madhu Instruments Pvt. Ltd., New Delhi, India). Strips were inserted in the lower fornix of each eye near the lateral canthus and left in place with the eyes closed. No topical anaesthetic was used. After 5 min, the strips were carefully removed, and the wetting length (WL) for both eyes was noted. The strips were individually packed in sample storage tubes, immediately frozen at − 20 °C, and transferred to − 80 °C within one week for further analysis. Previous history regarding eye diseases, eye medications, and the use of contact lenses was recorded.

### RNA Isolation

For RNA isolation, TF was initially eluted from the strips. For this, strips were cut into small pieces and wet with 40 µl RNAse-free water each. The tube containing the soaked pieces of the strips was placed in a bigger tube, and a hole was punched in it. Samples were centrifuged at 16.000 × g for 10 min.

Next, RNA was isolated using an adapted TRIzol-based protocol. In brief, TRIZol was added, and samples were incubated at room temperature for 5 min. 1-Bromo-3-Chlor-Propane was added to the samples, and the tubes were shaken for 20 s, followed by incubation at room temperature for 3 min. Phase separation was achieved via centrifugation at 12.000 × g at 4 °C for 15 min. The aqueous phase was transferred to a new tube. For precipitation, Glycoblue (Invitrogen, MA, USA) and Isopropanol were added, and samples were subsequently incubated at − 20 °C overnight. Samples were centrifuged at 12.000 × g at 4 °C for 30 min. The pellet was washed twice in 75% ethanol and dried to remove all ethanol.

For solubilization, the pellet was resuspended in RNAse-free water, and samples were shaken in an incubator at 55 °C for 2 min to facilitate resuspension. miRNA concentration was quantified using the Qubit miRNA assay (Invitrogen, MA, USA) and purity confirmed by spectrophotometry. Isolation was carried out for each sample separately with pooled stripes (left and right).

### miRNA RT-qPCR Screen

The QuantiMir Kit for the human miRNAome (SBI System Biosciences, CA, USA) was used to quantify miRNAs. This kit uses polyA-tailed miRNA real-time quantitative PCR (RT-qPCR) of 1113 miRNAs with 3 internal controls. The analysis was carried out in technical duplicates on 5 pooled samples: one containing all control samples, two containing 19 and 10, respectively, of the 29 total PD samples, as well as one containing all 10 PSP samples and one containing all 7 MSA samples. cDNA synthesis was carried out as indicated in the protocol on 500 ng of miRNA for each sample pool. For the RT-qPCR, the *Power* SYBR Green PCR Master Mix from Applied Biosystems (MA, USA) was used for all samples. Melting curve analysis was run for each plate to ensure the quality of the amplification reaction.

### Literature Search

We compared our data with previously published literature. For this, we searched PubMed for studies on miRNA as biomarkers in our disease groups. The following search query was employed and adapted for each disease: (microRNAs[Title/Abstract]) OR microRNA[Title/Abstract]) OR miRNA[Title/Abstract]) OR miRNAs[Title/Abstract]) OR MIR[Title/Abstract])) (biomarker [Title/Abstract] OR biomarkers [Title/Abstract]) AND PD/MSA/PSP. The diseases were searched for with the following addition to the query: (Parkinson’s disease[Title/Abstract] OR Parkinson’s[Title/Abstract]), (Progressive supranuclear palsy[Title/Abstract]), and (Multiple system atrophy[Title/Abstract]). Only studies reporting human data from either biofluids or brain tissue were considered. A complete listing of all used studies can be found in Table 2.

### Statistical and Data Analysis

Statistical analyses were performed using R version 4.4.2 (The R Foundation for Statistical Computing, Vienna, Austria). Data was plotted using the packages ggplot2 [40], pheatmap [41], and UpSetR [42]. The significance level was set at alpha = 0.05 (5%). For the overall cohort, categorical data were described by absolute and relative frequencies, and quantitative data by mean with standard deviation (SD) or median with minimum and maximum. The cumulative WL in mm/5 min for both eyes was calculated for each subject. To distinguish the mean values of WL between the different groups, multiple linear regression was performed, taking the confounders age and sex into account. Ordinary one-way ANOVA with Tukey post hoc testing was used to compare the distribution of relevant variables between groups (age, disease duration, miRNA concentration). Fisher’s exact test was used for categorical variables (sex, eye diseases, eye medication). Pearson’s correlation coefficient was used to estimate the association between miRNA concentration and clinical data. For relevant effect measures, 95% confidence intervals were calculated.

For the miRNA quantitative data, raw cycle threshold (CT) values of miRNAs were categorized according to the probability of expression: if both technical replicates had a CT value of < 40 and an SD of < 5, miRNAs were considered amplified with high certainty. All other miRNAs were divided into two groups for the overall expression analysis: miRNAs not amplified in both technical replicates were considered not amplified, and any condition in between was considered amplified with low certainty. Both of these groups were summarized for the intersection analysis and the comparison to literature. For the two PD biological replicates, any miRNA that was considered amplified with high certainty according to these criteria in at least one biological replicate was considered amplified with high certainty.

miRNA names were converted using the miRBaseConverter package. For the identification of miRNAs present in brain tissue, the DIANA-miTED tissue expression database was used [43]. Settings to identify all miRNAs detected in healthy controls in brain or brain stem tissue were used. An expression value of > 1 read counts was considered expressed. For the over-representation analysis (ORA), we used the DIANA miRPath online tool (v4) [44]. Standard settings were used: miRTarBase annotated targets without long non-coding targets were used in the Gene Union algorithm with a p-value threshold of 0.05 and FDR correction. The resulting lists were further filtered for terms that included targets from 5 or more miRNAs and had an FDR < 0.01. In addition, REVIGO [45] was employed for clustering analysis of the list of terms. This tool allows GO term clustering by hierarchy using semantic similarity, p-adjusted values, and term proximity measures. The default settings for a reduction to a small size were used, and the FDR of the terms was added as additional information.

## Supplementary Information

Below is the link to the electronic supplementary material.

Supplementary file1 (DOCX 1.15 MB)

Supplementary file2 (XLS 224 KB)

## Data Availability

Original data is available with the investigators upon reasonable request.
