# Supplementary material for: An Exploratory Analysis of Differential Tear Fluid miRNAs in Patients with Parkinson’s Disease and Atypical Parkinsonian Syndromes
Source: Mol Neurobiol. 2025 Aug 4;62(12):16397–409. doi: 10.1007/s12035-025-05252-2 (PMC12559130; doi:10.1007/s12035-025-05252-2)
Supplement: Supplementary file 1 — Supplementary file1 (DOCX 1.15 MB) [file 12035_2025_5252_MOESM1_ESM.docx]

**Supplementary Material**

**Differential tear fluid miRNAs in patients with Parkinson’s disease and atypical Parkinsonian syndromes**

Antonia F. Demleitner^1^*, Lucas Caldi Gomes^1^, Lara Wenz^1^, Laura Tzeplaeff^1^, Dominik Pürner^1^, Elena Luib^1^, Lea H. Kunze^1^, Paul Lingor^1,2,3^

^1^Department of Neurology, Klinikum rechts der Isar, TUM Universitätsklinikum, School of Medicine and Health, Technical University of Munich, Munich, Germany

^2^ DZNE, German Center for Neurodegenerative Diseases, Munich, Germany

^3^ Munich Cluster for Systems Neurology (SyNergy), Munich Germany

*Corresponding Author: Paul Lingor, paul.lingor@tum.de, Department of Neurology, Klinikum rechts der Isar, TUM Universitätsklinikum, School of Medicine and Health, Technical University of Munich, Ismaninger Str. 22, 81675 Munich


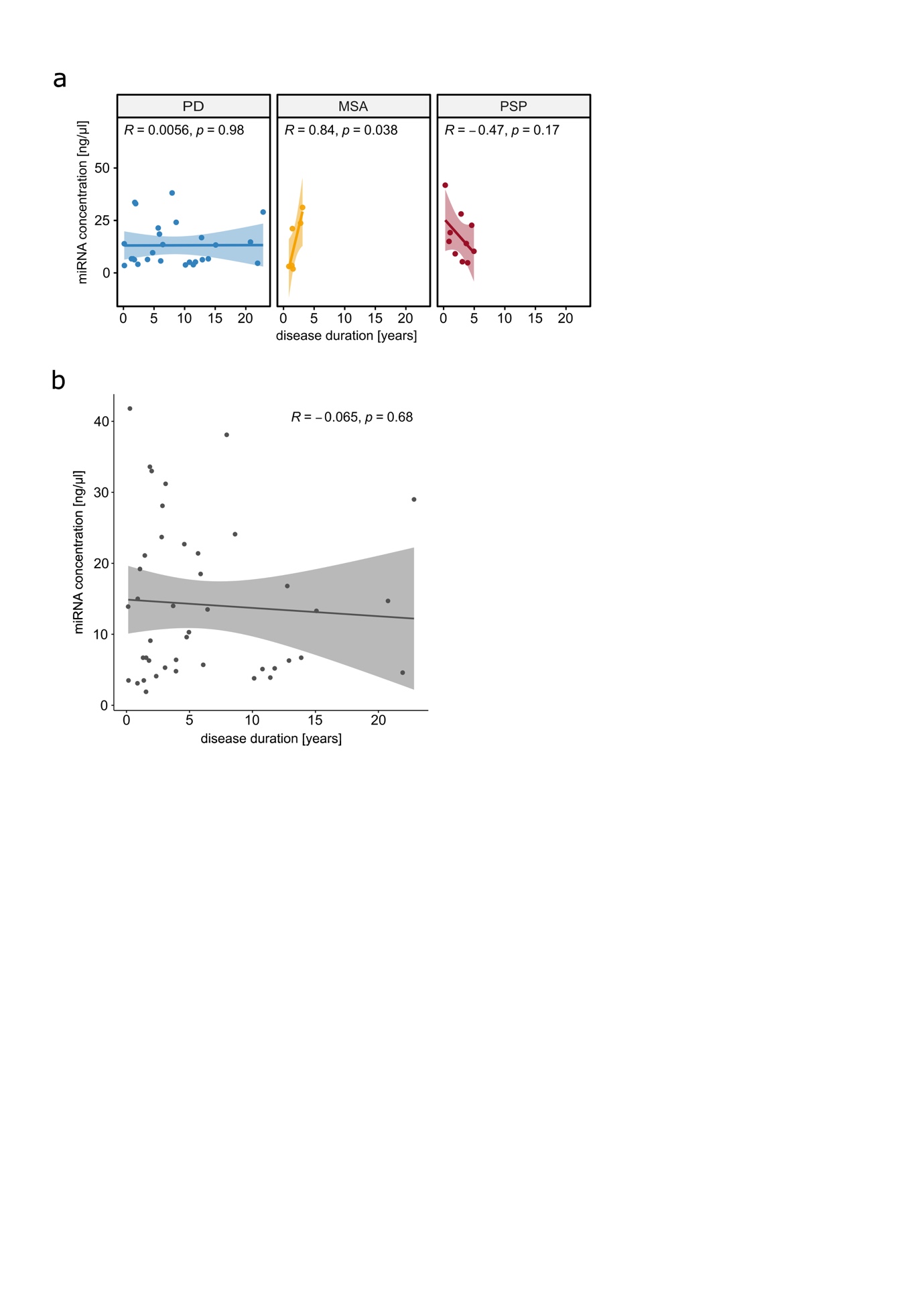


**Supplementary Fig. S1 Correlation of miRNA concentration in tear fluid with disease duration**

a) Correlation of miRNA concentration and disease duration within the subgroups of PD (blue), MSA (green) and PSP (red). b) Correlation of miRNA concentration and disease duration in a cohort of all disease groups. PD = Parkinson’s disease, MSA = multiple system atrophy, PSP = progressive supranuclear palsy. Pearson’s correlation (Coefficient = R) was used for analyses. Data are depicted as a regression line with 95% confidence interval and individual data points.


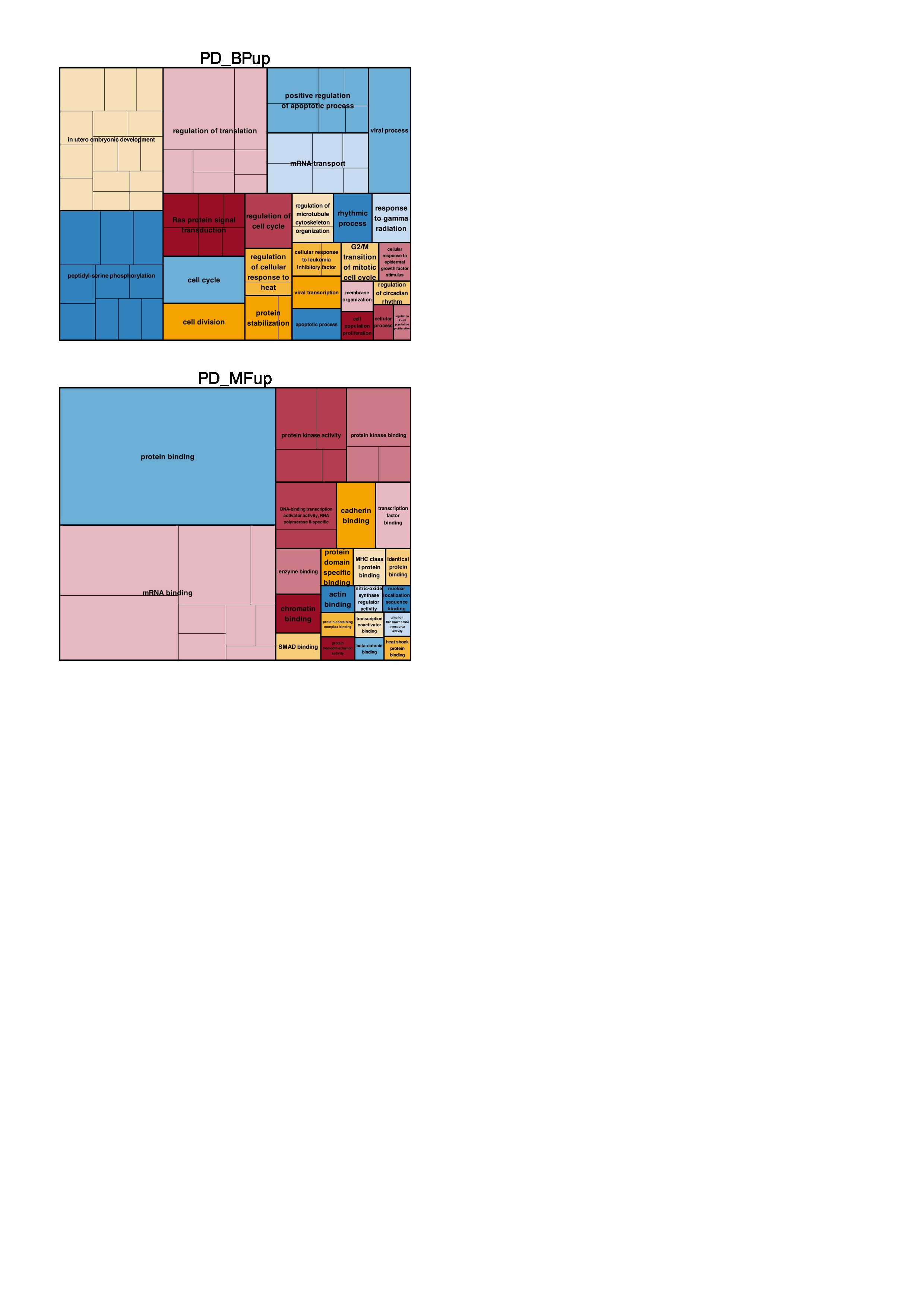


**Supplementary Fig. 2** **REVIGO Treemap Plots of all significant GO terms for the PD intersections**

Labeling was determined by semantic similarity analysis. These representatives are combined into "superclusters", representing loosely related terms and visualized using different colors. The size of the clusters is adjusted to reflect the P-value and frequency of the GO term in the Homo sapiens GOA database. BP = Biological process, MF = Molecular function, PD = Parkinson’s disease.

###

###

###
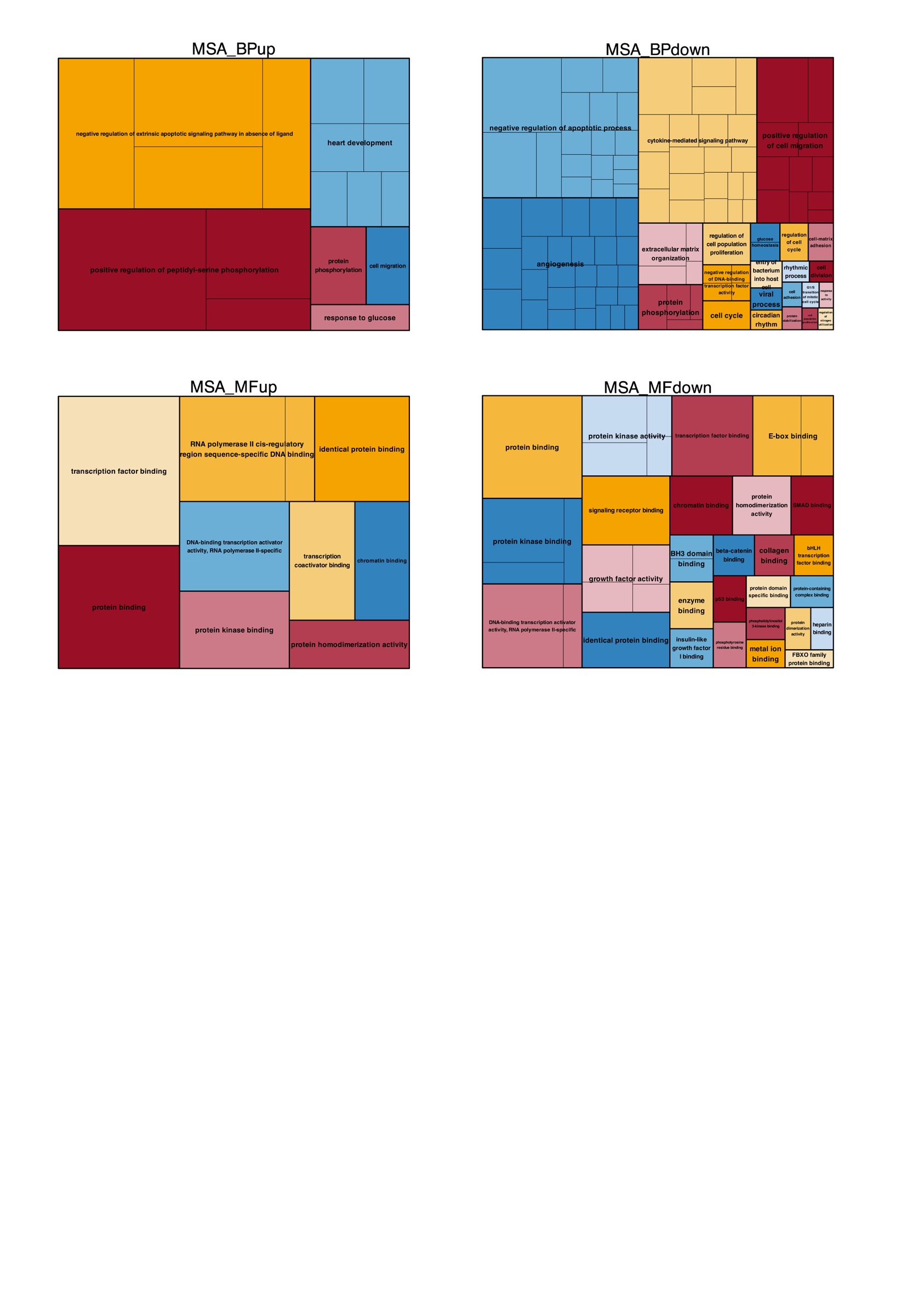


**Supplementary Fig. 3** **REVIGO Treemap Plots of all significant GO terms for the MSA intersections**

Labeling was determined by semantic similarity analysis. These representatives are combined into "superclusters", representing loosely related terms and visualized using different colors. The size of the clusters is adjusted to reflect the P-value and frequency of the GO term in the Homo sapiens GOA database. BP = Biological process, MF = Molecular function, MSA = multiple system atrophy.

###
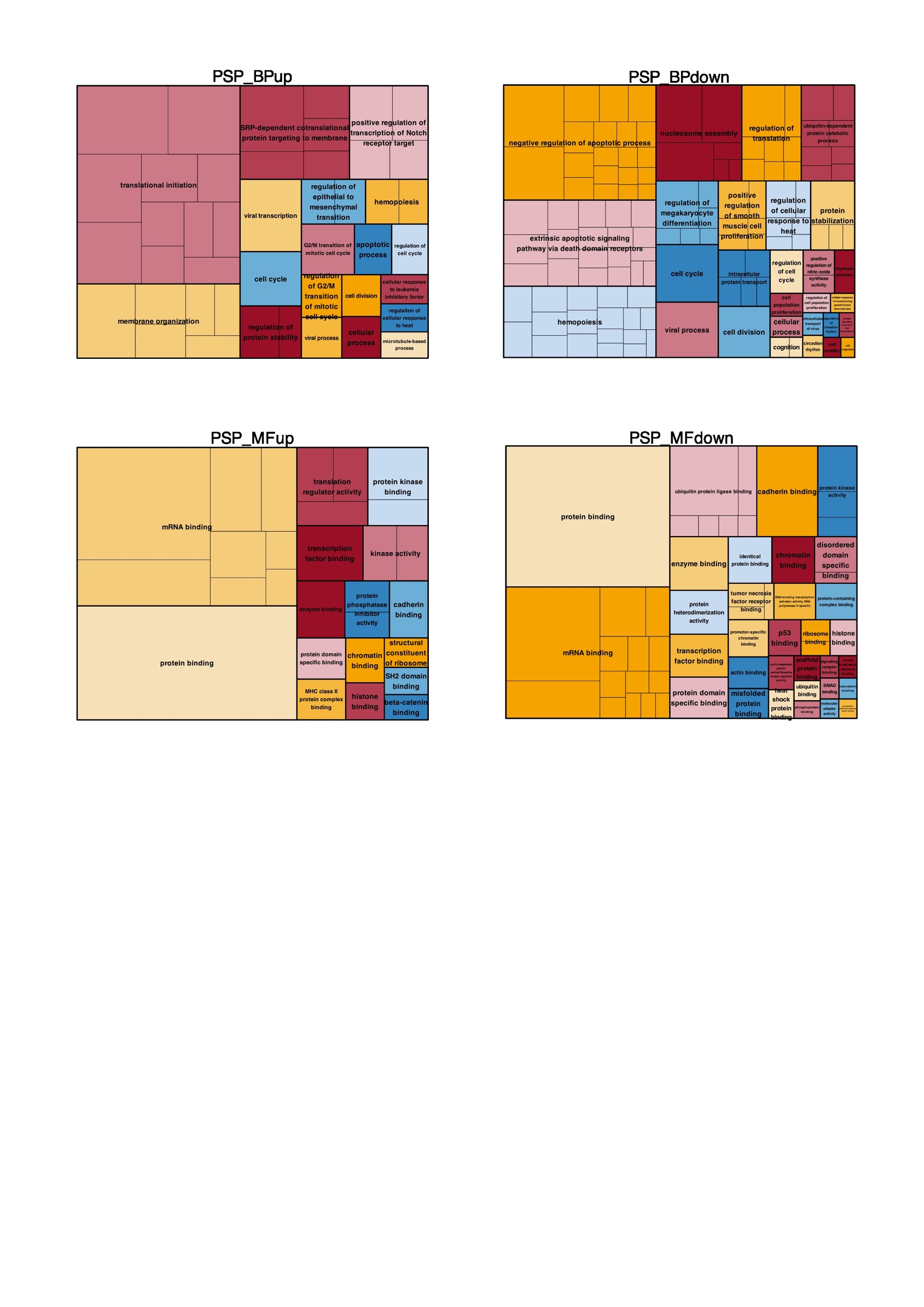


**Supplementary Fig. 4** **REVIGO Treemap Plots of all significant GO terms for the PSP intersections**

Labeling was determined by semantic similarity analysis. These representatives are combined into "superclusters", representing loosely related terms and visualized using different colors. The size of the clusters is adjusted to reflect the P-value and frequency of the GO term in the Homo sapiens GOA database. BP = Biological process, MF = Molecular function, PSP = progressive supranuclear palsy.

###

| Intersection | Name in QuantiMir Screen | Accession number | miRBase v22 Name |
| --- | --- | --- | --- |
| found in PD | hsa-miR-517c-5p | not listed | not listed |
| found in PD | hsa-miR-130a-5p | MIMAT0004593 | hsa-miR-130a-5p |
| found in PD | hsa-miR-20b-3p | MIMAT0004752 | hsa-miR-20b-3p |
| found in PD | hsa-miR-182-3p | MIMAT0000260 | hsa-miR-182-3p |
| found in PD | hsa-miR-192-3p | MIMAT0004543 | hsa-miR-192-3p |
| found in PD | hsa-miR-106b-3p | MIMAT0004672 | hsa-miR-106b-3p |
| found in PD | hsa-miR-30d-3p | MIMAT0004551 | hsa-miR-30d-3p |
| found in PD | hsa-miR-454-5p | MIMAT0003884 | hsa-miR-454-5p |
| found in PD | hsa-let-7d-3p | MIMAT0004484 | hsa-let-7d-3p |
| found in PD | hsa-miR-377 | MIMAT0000730 | hsa-miR-377-3p |
| found in PD | hsa-miR-516a-5p | MIMAT0004770 | hsa-miR-516a-5p |
| found in PD | hsa-miR-410 | MIMAT0002171 | hsa-miR-410-3p |
| found in PD | hsa-miR-567 | MIMAT0003231 | hsa-miR-567 |
| found in PD | hsa-miR-573 | MIMAT0003238 | hsa-miR-573 |
| found in PD | hsa-miR-500b | MIMAT0016925 | hsa-miR-500b-5p |
| found in PD | hsa-miR-27a-5p | MIMAT0004501 | hsa-miR-27a-5p |
| found in PD | hsa-miR-517b | MIMAT0002857 | hsa-miR-517b-3p |
| found in PD | hsa-miR-3191 | MIMAT0015075 | hsa-miR-3191-3p |
| found in PD | hsa-miR-505 | MIMAT0002876 | hsa-miR-505-3p |
| found in PD | hsa-miR-3124 | MIMAT0014986 | hsa-miR-3124-5p |
| found in PD | hsa-miR-524-5p | MIMAT0002849 | hsa-miR-524-5p |
| found in PD | hsa-miR-518d-3p | MIMAT0002864 | hsa-miR-518d-3p |
| found in PD | hsa-miR-196b | MIMAT0001080 | hsa-miR-196b-5p |
| found in PD | hsa-miR-103 | MIMAT0000101 | hsa-miR-103a-3p |
| found in PD | hsa-miR-3198 | MIMAT0015083 | hsa-miR-3198 |
| found in PD | hsa-miR-4288 | MIMAT0016918 | hsa-miR-4288 |
| found in PD | hsa-miR-298 | MIMAT0004901 | hsa-miR-298 |
| found in PD | hsa-miR-199a-5p | MIMAT0000231 | hsa-miR-199a-5p |
| found in PD | hsa-miR-542-5p | MIMAT0003340 | hsa-miR-542-5p |
| found in PD | hsa-miR-328 | MIMAT0000752 | hsa-miR-328-3p |
| found in PD | hsa-miR-188-3p | MIMAT0004613 | hsa-miR-188-3p |
| found in PD | hsa-miR-374a | MIMAT0000727 | hsa-miR-374a-5p |
| found in PD | hsa-miR-654-5p | MIMAT0003330 | hsa-miR-654-5p |
| found in PD | hsa-miR-597 | MIMAT0003265 | hsa-miR-597-5p |
| found in PD | hsa-miR-596 | MIMAT0003264 | hsa-miR-596 |
| found in PD | hsa-miR-3172 | not listed | not listed |
| found in PD | hsa-miR-193a-3p | MIMAT0000459 | hsa-miR-193a-3p |
| found in PD | hsa-miR-670 | MIMAT0010357 | hsa-miR-670-5p |
| found in PD | hsa-miR-508-5p | MIMAT0004778 | hsa-miR-508-5p |
| found in PD | hsa-miR-4260 | MIMAT0016881 | hsa-miR-4260 |
| found in PD | hsa-miR-548v | MIMAT0015020 | hsa-miR-548v |
| found in PD | hsa-miR-671-5p | MIMAT0003880 | hsa-miR-671-5p |
| found in PD | hsa-miR-1909 | MIMAT0007883 | hsa-miR-1909-3p |
| found in PD | hsa-miR-3149 | MIMAT0015022 | hsa-miR-3149 |
| found in PD | hsa-miR-183 | MIMAT0000261 | hsa-miR-183-5p |
| found in PD | hsa-miR-455-3p | MIMAT0004784 | hsa-miR-455-3p |
| found in PD | hsa-miR-522 | MIMAT0002868 | hsa-miR-522-3p |
| found in PD | hsa-miR-363 | MIMAT0000707 | hsa-miR-363-3p |
| found in PD | hsa-miR-142-5p | MIMAT0000433 | hsa-miR-142-5p |
| found in PD | hsa-miR-487b | MIMAT0003180 | hsa-miR-487b-3p |
| found in PD | hsa-miR-938 | MIMAT0004981 | hsa-miR-938 |
| found in PD | hsa-miR-4290 | MIMAT0016921 | hsa-miR-4290 |
| found in PD | hsa-miR-4279 | MIMAT0016909 | hsa-miR-4279 |
| found in PD | hsa-miR-128 | MIMAT0000424 | hsa-miR-128-3p |
| found in PD | hsa-miR-876-5p | MIMAT0004924 | hsa-miR-876-5p |
| not found in PD | hsa-miR-193b-5p | MIMAT0004767 | hsa-miR-193b-5p |
| not found in PD | hsa-miR-95 | MIMAT0000094 | hsa-miR-95-3p |
| not found in PD | hsa-miR-886-5p | not listed | not listed |
| not found in PD | hsa-miR-934 | MIMAT0004977 | hsa-miR-934 |
| found in PSP | hsa-miR-488-5p | MIMAT0002804 | hsa-miR-488-5p |
| found in PSP | hsa-miR-18a-3p | MIMAT0002891 | hsa-miR-18a-3p |
| found in PSP | hsa-miR-380-5p | MIMAT0000734 | hsa-miR-380-5p |
| found in PSP | hsa-miR-148a-5p | MIMAT0004549 | hsa-miR-148a-5p |
| found in PSP | hsa-miR-33a-3p | MIMAT0004506 | hsa-miR-33a-3p |
| found in PSP | hsa-miR-432 | MIMAT0002814 | hsa-miR-432-5p |
| found in PSP | hsa-miR-29c-5p | MIMAT0004673 | hsa-miR-29c-5p |
| found in PSP | hsa-miR-27b-5p | MIMAT0004588 | hsa-miR-27b-5p |
| found in PSP | hsa-miR-2117 | MIMAT0011162 | hsa-miR-2117 |
| found in PSP | hsa-miR-558 | MIMAT0003222 | hsa-miR-558 |
| found in PSP | hsa-miR-423-5p | MIMAT0004748 | hsa-miR-423-5p |
| found in PSP | hsa-miR-138-2* | MIMAT0004596 | hsa-miR-138-2-3p |
| found in PSP | hsa-miR-4311 | MIMAT0016863 | hsa-miR-4311 |
| found in PSP | hsa-miR-1302 | MIMAT0005890 | hsa-miR-1302 |
| found in PSP | hsa-let-7f-1* | MIMAT0004486 | hsa-let-7f-1-3p |
| found in PSP | hsa-miR-1293 | MIMAT0005883 | hsa-miR-1293 |
| found in PSP | hsa-let-7 | not listed | not listed |
| found in PSP | hsa-miR-517a | MIMAT0002852 | hsa-miR-517a-3p |
| found in PSP | hsa-miR-532-5p | MIMAT0002888 | hsa-miR-532-5p |
| found in PSP | hsa-miR-643 | MIMAT0003313 | hsa-miR-643 |
| found in PSP | hsa-miR-521 | MIMAT0002854 | hsa-miR-521 |
| found in PSP | hsa-miR-4301 | MIMAT0016850 | hsa-miR-4301 |
| found in PSP | hsa-miR-1272 | MIMAT0005925 | hsa-miR-1272 |
| found in PSP | hsa-miR-3121 | MIMAT0014983 | hsa-miR-3121-3p |
| found in PSP | hsa-miR-1248 | MIMAT0005900 | hsa-miR-1248 |
| found in PSP | hsa-miR-1227 | MIMAT0005580 | hsa-miR-1227-3p |
| found in PSP | hsa-let-7-2* | not listed | not listed |
| found in PSP | hsa-miR-502-3p | MIMAT0004775 | hsa-miR-502-3p |
| found in PSP | hsa-miR-2116 | MIMAT0011160 | hsa-miR-2116-5p |
| found in PSP | hsa-miR-425 | MIMAT0003393 | hsa-miR-425-5p |
| found in PSP | hsa-miR-764 | MIMAT0010367 | hsa-miR-764 |
| found in PSP | hsa-miR-340 | MIMAT0004692 | hsa-miR-340-5p |
| found in PSP | hsa-miR-181a-2* | MIMAT0004558 | hsa-miR-181a-2-3p |
| found in PSP | hsa-miR-4291 | MIMAT0016922 | hsa-miR-4291 |
| found in PSP | hsa-miR-194 | MIMAT0000460 | hsa-miR-194-5p |
| not found in PSP | hsa-miR-10b-3p | MIMAT0004556 | hsa-miR-10b-3p |
| not found in PSP | hsa-miR-223 | MIMAT0000280 | hsa-miR-223-3p |
| not found in PSP | hsa-miR-132 | MIMAT0000426 | hsa-miR-132-3p |
| not found in PSP | hsa-miR-27b | MIMAT0000419 | hsa-miR-27b-3p |
| not found in PSP | hsa-miR-202 | MIMAT0002811 | hsa-miR-202-3p |
| not found in PSP | hsa-miR-23b | MIMAT0000418 | hsa-miR-23b-3p |
| not found in PSP | hsa-miR-151-3p | MIMAT0000757 | hsa-miR-151a-3p |
| not found in PSP | hsa-miR-23a | MIMAT0000078 | hsa-miR-23a-3p |
| not found in PSP | hsa-let-7c | MIMAT0000064 | hsa-let-7c-5p |
| not found in PSP | hsa-miR-523 | MIMAT0002840 | hsa-miR-523-3p |
| not found in PSP | hsa-miR-488 | MIMAT0004763 | hsa-miR-488-3p |
| not found in PSP | hsa-miR-222 | MIMAT0000279 | hsa-miR-222-3p |
| not found in PSP | hsa-miR-206 | MIMAT0000462 | hsa-miR-206 |
| not found in PSP | hsa-miR-3164 | MIMAT0015038 | hsa-miR-3164 |
| not found in PSP | hsa-miR-559 | MIMAT0003223 | hsa-miR-559 |
| not found in PSP | hsa-miR-618 | MIMAT0003287 | hsa-miR-618 |
| not found in PSP | hsa-miR-219-1-3p | MIMAT0004567 | hsa-miR-219a-1-3p |
| not found in PSP | hsa-miR-520d-3p | MIMAT0002856 | hsa-miR-520d-3p |
| not found in PSP | hsa-miR-192 | MIMAT0000222 | hsa-miR-192-5p |
| not found in PSP | hsa-miR-4266 | MIMAT0016892 | hsa-miR-4266 |
| not found in PSP | hsa-miR-1270 | MIMAT0005924 | hsa-miR-1270 |
| not found in PSP | hsa-miR-490-5p | MIMAT0004764 | hsa-miR-490-5p |
| not found in PSP | hsa-miR-34a | MIMAT0000255 | hsa-miR-34a-5p |
| not found in PSP | hsa-miR-524-3p | MIMAT0002850 | hsa-miR-524-3p |
| not found in PSP | hsa-miR-503 | MIMAT0002874 | hsa-miR-503-5p |
| not found in PSP | hsa-miR-539 | MIMAT0003163 | hsa-miR-539-5p |
| not found in PSP | hsa-miR-99b | MIMAT0000689 | hsa-miR-99b-5p |
| not found in PSP | hsa-miR-10b-3p | MIMAT0004556 | hsa-miR-10b-3p |
| not found in PSP | hsa-miR-223 | MIMAT0000280 | hsa-miR-223-3p |
| not found in PSP | hsa-miR-132 | MIMAT0000426 | hsa-miR-132-3p |
| not found in PSP | hsa-miR-27b | MIMAT0000419 | hsa-miR-27b-3p |
| not found in PSP | hsa-miR-202 | MIMAT0002811 | hsa-miR-202-3p |
| not found in PSP | hsa-miR-23b | MIMAT0000418 | hsa-miR-23b-3p |
| not found in PSP | hsa-miR-151-3p | MIMAT0000757 | hsa-miR-151a-3p |
| not found in PSP | hsa-miR-23a | MIMAT0000078 | hsa-miR-23a-3p |
| not found in PSP | hsa-let-7c | MIMAT0000064 | hsa-let-7c-5p |
| not found in PSP | hsa-miR-523 | MIMAT0002840 | hsa-miR-523-3p |
| not found in PSP | hsa-miR-488 | MIMAT0004763 | hsa-miR-488-3p |
| not found in PSP | hsa-miR-222 | MIMAT0000279 | hsa-miR-222-3p |
| not found in PSP | hsa-miR-206 | MIMAT0000462 | hsa-miR-206 |
| not found in PSP | hsa-miR-3164 | MIMAT0015038 | hsa-miR-3164 |
| not found in PSP | hsa-miR-559 | MIMAT0003223 | hsa-miR-559 |
| not found in PSP | hsa-miR-618 | MIMAT0003287 | hsa-miR-618 |
| not found in PSP | hsa-miR-219-1-3p | MIMAT0004567 | hsa-miR-219a-1-3p |
| not found in PSP | hsa-miR-520d-3p | MIMAT0002856 | hsa-miR-520d-3p |
| not found in PSP | hsa-miR-192 | MIMAT0000222 | hsa-miR-192-5p |
| not found in PSP | hsa-miR-4266 | MIMAT0016892 | hsa-miR-4266 |
| not found in PSP | hsa-miR-1270 | MIMAT0005924 | hsa-miR-1270 |
| not found in PSP | hsa-miR-490-5p | MIMAT0004764 | hsa-miR-490-5p |
| not found in PSP | hsa-miR-34a | MIMAT0000255 | hsa-miR-34a-5p |
| not found in PSP | hsa-miR-524-3p | MIMAT0002850 | hsa-miR-524-3p |
| not found in PSP | hsa-miR-503 | MIMAT0002874 | hsa-miR-503-5p |
| not found in PSP | hsa-miR-539 | MIMAT0003163 | hsa-miR-539-5p |
| not found in PSP | hsa-miR-99b | MIMAT0000689 | hsa-miR-99b-5p |
| found in MSA | hsa-miR-374b-3p | MIMAT0004956 | hsa-miR-374b-3p |
| found in MSA | hsa-miR-129-3-3p | not listed | not listed |
| found in MSA | hsa-miR-3142 | MIMAT0015011 | hsa-miR-3142 |
| found in MSA | hsa-miR-4330 | MIMAT0016924 | hsa-miR-4330 |
| found in MSA | hsa-miR-22 | MIMAT0000077 | hsa-miR-22-3p |
| found in MSA | hsa-miR-130a | MIMAT0000425 | hsa-miR-130a-3p |
| found in MSA | hsa-miR-1182 | MIMAT0005827 | hsa-miR-1182 |
| found in MSA | hsa-miR-532-3p | MIMAT0004780 | hsa-miR-532-3p |
| found in MSA | hsa-miR-608 | MIMAT0003276 | hsa-miR-608 |
| found in MSA | hsa-miR-323-3p | MIMAT0000755 | hsa-miR-323a-3p |
| found in MSA | hsa-miR-4320 | MIMAT0016871 | hsa-miR-4320 |
| found in MSA | hsa-miR-15a | MIMAT0000068 | hsa-miR-15a-5p |
| found in MSA | hsa-miR-765 | MIMAT0003945 | hsa-miR-765 |
| found in MSA | hsa-miR-566 | not listed | not listed |
| not found in MSA | hsa-miR-1226-5p | MIMAT0005576 | hsa-miR-1226-5p |
| not found in MSA | hsa-miR-1914-3p | MIMAT0007890 | hsa-miR-1914-3p |
| not found in MSA | hsa-miR-1909-5p | MIMAT0007882 | hsa-miR-1909-5p |
| not found in MSA | hsa-miR-24-1* | MIMAT0000079 | hsa-miR-24-1-5p |
| not found in MSA | hsa-miR-3138 | MIMAT0015006 | hsa-miR-3138 |
| not found in MSA | hsa-miR-1537 | MIMAT0007399 | hsa-miR-1537-3p |
| not found in MSA | hsa-miR-429 | MIMAT0001536 | hsa-miR-429 |
| not found in MSA | hsa-miR-211 | MIMAT0000268 | hsa-miR-211-5p |
| not found in MSA | hsa-miR-342-3p | MIMAT0000753 | hsa-miR-342-3p |
| not found in MSA | hsa-miR-29c-3p | MIMAT0000681 | hsa-miR-29c-3p |
| not found in MSA | hsa-miR-4313 | MIMAT0016865 | hsa-miR-4313 |
| not found in MSA | hsa-miR-204 | MIMAT0000265 | hsa-miR-204-5p |
| not found in MSA | hsa-miR-382 | MIMAT0000737 | hsa-miR-382-5p |
| not found in MSA | hsa-miR-216a | MIMAT0000273 | hsa-miR-216a-5p |
| not found in MSA | hsa-miR-34b | MIMAT0004676 | hsa-miR-34b-3p |
| not found in MSA | hsa-miR-3183 | MIMAT0015063 | hsa-miR-3183 |
| not found in MSA | hsa-miR-1180 | MIMAT0005825 | hsa-miR-1180-3p |
| not found in MSA | hsa-miR-1275 | MIMAT0005929 | hsa-miR-1275 |
| not found in MSA | hsa-miR-502-5p | MIMAT0002873 | hsa-miR-502-5p |
| not found in MSA | hsa-miR-93 | MIMAT0000093 | hsa-miR-93-5p |
| not found in MSA | hsa-miR-4309 | MIMAT0016859 | hsa-miR-4309 |
| not found in MSA | hsa-miR-4274 | MIMAT0016906 | hsa-miR-4274 |
| not found in MSA | hsa-miR-330-3p | MIMAT0000751 | hsa-miR-330-3p |
| not found in MSA | hsa-miR-3137 | MIMAT0015005 | hsa-miR-3137 |
| not found in MSA | hsa-miR-661 | MIMAT0003324 | hsa-miR-661 |
| not found in MSA | hsa-miR-631 | MIMAT0003300 | hsa-miR-631 |
| not found in MSA | hsa-miR-152 | MIMAT0000438 | hsa-miR-152-3p |
| not found in MSA | hsa-miR-622 | MIMAT0003291 | hsa-miR-622 |
| not found in MSA | hsa-miR-1258 | MIMAT0005909 | hsa-miR-1258 |
| not found in MSA | hsa-miR-1253 | MIMAT0005904 | hsa-miR-1253 |
| not found in MSA | hsa-miR-4315 | MIMAT0016866 | hsa-miR-4315 |
| not found in MSA | hsa-miR-3135 | MIMAT0015001 | hsa-miR-3135a |
| not found in MSA | hsa-let-7e | MIMAT0000066 | hsa-let-7e-5p |
| not found in MSA | hsa-miR-1281 | MIMAT0005939 | hsa-miR-1281 |
| not found in MSA | hsa-miR-632 | MIMAT0003302 | hsa-miR-632 |
| not found in MSA | hsa-miR-214-3p | MIMAT0000271 | hsa-miR-214-3p |
| not found in MSA | hsa-miR-448 | MIMAT0001532 | hsa-miR-448 |
| not found in MSA | hsa-miR-1265 | MIMAT0005918 | hsa-miR-1265 |
| not found in MSA | hsa-miR-1203 | MIMAT0005866 | hsa-miR-1203 |
| not found in MSA | hsa-miR-3175 | MIMAT0015052 | hsa-miR-3175 |
| not found in MSA | hsa-miR-92a-1* | MIMAT0004507 | hsa-miR-92a-1-5p |

**Supplementary Table S1 Overview over the miRNAs in the intersections of Figure 2B**

Shown are their annotation in the QuantiMir kit as well as their respective accession number and their annotation in the v22 version of miRBase. miRNA names were converted using the miRBaseConverter package within the BiocManager package (v1.30.0) in R. PD = Parkinson’s disease, MSA = multiple system atrophy, PSP = progressive supranuclear palsy.

**Supplementary Data S2** **Overview over the GO terms summarized by REVIGO**

Summarization uses LogSize, Frequency, Uniqueness and Dispensability measures. Separate sheets show data for each condition: exclusively found (“-UP”) or exclusively absent (“-DOWN”) miRNAs of the different disease groups for the BP and MF terms. BP = Biological process, MF = Molecular function, PD = Parkinson’s disease, MSA = multiple system atrophy, PSP = progressive supranuclear palsy.
